# Feasibility of non-invasive neuromonitoring using BIS and NIRS during endovascular treatment of acute ischemic stroke

**DOI:** 10.1186/s42466-025-00420-0

**Published:** 2025-08-26

**Authors:** David Batra, Min Chen, Jan Meis, Markus A. Möhlenbruch, Christina Klose, Peter Ringleb, Vishank Shah, Julian Bösel, Silvia Schönenberger

**Affiliations:** 1 Department of Neurology, Heidelberg University Hospital, Heidelberg, Germany; 2 Institute of Medical Biometry and Informatics, University of Heidelberg, Heidelberg, Germany; 3 Department of Neuroradiology, Heidelberg University Hospital, Heidelberg, Germany; 4 Department of Neurology, Anesthesiology and Critical Care Medicine, Johns Hopkins University School of Medicine, Baltimore, MD USA; 5 Department of Neurology, Friedrich-Ebert-Hospital, Neumünster, Germany

## Abstract

**Background:**

Endovascular thrombectomy (EVT)—often combined with intravenous thrombolysis—is the standard of care for acute ischemic stroke (AIS) secondary to large vessel occlusions (LVO). While indications keep expanding, the feasibility and utility of intra-procedural neuromonitoring of the sedated patient has neither been clarified nor characterized.

**Objective:**

To evaluate the feasibility of near-infrared spectroscopy (NIRS) for cortical oxygenation and bispectral index (BIS) for electroencephalographic function as non-invasive neuromonitoring tools for AIS patients undergoing EVT, and assess their utility in predicting successful recanalization.

**Methods:**

We extracted data on all patients receiving continuous NIRS and/or BIS monitoring in the Sedation versus Intubation for Endovascular Stroke TreAtment (SIESTA) clinical trial. SIESTA randomized AIS patients undergoing EVT for anterior proximal LVO to general anesthesia versus conscious sedation. For this analysis, the primary outcomes included changes in NIRS and BIS values pre- and post-recanalization and associations of parameter changes with successful or unsuccessful recanalization outcomes. Statistical analysis was performed using a Wilcoxon signed rank tests.

**Results:**

Of the 150 patients, 66 were monitored continuously with NIRS, and 50 with BIS. An increased NIRS-derived cerebral tissue oxygenation (stated as tissue saturation index – TSI) was observed in affected hemisphere following successful recanalization, as well as a significant reduction in the difference between affected and unaffected hemispheres. In contrast, no significant changes were observed with BIS monitoring between pre- and post-recanalization status.

**Conclusion:**

In this post-hoc analysis, changes in NIRS monitoring were associated with successful reperfusion. Non-invasive oximetry by NIRS may serve as a valuable tool during and after mechanical thrombectomy to detect and respond to an insufficient perfusion or re-occlusion.

## Introduction

Intravenous thrombolysis and mechanical recanalization with endovascular thrombectomy (EVT) have significantly changed the landscape of acute stroke care in the past decade [1–6]. The indications for EVT in acute ischemic stroke (AIS) patients with large vessel occlusions (LVO) continue to expand to now include longer time windows (up to 24 h post-ictus), medium to distal vessel occlusions and most recently, large infarct cores [7–16]. Although overall a safe procedure, expanding indications place patients undergoing EVT at a higher risk for intraprocedural complications such as distal thrombus migration. Establishing a standardized, non-invasive method of neuromonitoring during EVT, that is most often carried out in the sedated or anesthetized patient, appears desirable to promptly detect successful reperfusion. However, while systemic hemodynamic monitoring such as for blood pressure (BP), heart rate and rhythm (electrocardiogram [ECG]) and oxygen saturation (SpO_2_) appear to be periprocedural standard [17, 18], the role neuromonitoring has hardly been clarified.

One common method of neuromonitoring during cardiovascular, neurosurgical and pediatric surgical procedures is near-infrared spectroscopy (NIRS), which measures regional cerebral oxygen saturation a few centimeters beneath the skull, thus allowing determination in both cortical and subcortical areas [19, 20]. NIRS is a non-invasive, easy-to-perform, and reproducible bedside method to assess superficial cerebral oxygenation, serving as a surrogate marker of cerebral blood flow (CBF). The recorded NIRS values in this study are presented as tissue saturation index derived from oxy- and deoxyhemoglobin. NIRS monitoring involves placement of adhesive optodes to the forehead of the patient. These optodes contain a light source on the patient-facing surface that emits near-infrared light and two detectors that measure the transmission-absorption ratio according to the Lambert–Beer law and hence depending on the oxygen levels of the blood. In contrast to the pulsatile measurement of a pulse oximeter, NIRS measurement is continuous. As a result, it cannot differentiate between arterial and venous blood flow but instead reflects the balance between oxygen consumption and supply [21].

Another non-invasive method of neuromonitoring, the Bispectral Index (BIS), mainly used to measure anesthesia depth in the operation room (OR), is based on electroencephalogram (EEG) recordings of frontal brain activity [22]. BIS monitoring involves a singular frontal EEG lead that is translated into a dimensionless number between 0 and 100 through a proprietary algorithm, representing a spectrum from complete wakefulness (BIS = 100) to complete absence of brain activity (BIS = 0) [23]. The EEG components incorporated into the algorithm include the frequency below which 95% of the power spectrum resides, the relative beta ratio, the relative synchrony of fast and slow waves, and the burst-suppression ratio [24].

In previous studies evaluating non-invasive neuromonitoring in AIS, NIRS has been predominantly used [21, 25, 26]. BIS has not been systematically studied in AIS settings, although it has been shown to potentially indicate delayed cerebral ischemia (DCI) in cases of subarachnoid hemorrhage [23].

In the single-center randomized clinical trial, Sedation vs. Intubation for endovascular stroke treatment (SIESTA) [27], which compared different sedation strategies in AIS patients undergoing EVT, both NIRS and BIS monitoring was performed as part of a standardized intra-procedural monitoring protocol. We sought to examine the feasibility and utility of and NIRS and BIS monitoring during EVT after AIS. We hypothesized that these methods could potentially detect an AIS caused by LVOs and reliably indicate the restoration of perfusion as well as possible complications including intracranial hemorrhage or thrombus migration post-EVT. Secondarily, we also sought to investigate whether there was a measurable impact of the chosen sedation method on the NIRS and BIS indices.

## Methods

### Data source and patients

The SIESTA trial [27] enrolled 150 AIS patients with a National Institutes of Health Stroke Scale (NIHSS) score greater than 10, LVO of the anterior circulation involving the internal carotid artery (ICA), the M1 or M2 segment of the middle cerebral artery (MCA) and those received either intravenous thrombolytic therapy with tPA in combination with EVT or EVT alone. Patients were randomized to receive either general anesthesia with intubation or conscious sedation during EVT. The trial did not show improvement in primary outcome of early neurological improvement (NIHSS improvement of ≥ 4-points) with conscious sedation over general anesthesia. In this secondary analysis we included all patients enrolled in the SIESTA trial that received NIRS and/or BIS monitoring during EVT.

### BIS/NIRS monitoring protocol

Upon arrival in the angiography suite, a BIS electrode was placed on the patient’s forehead, and NIRS optodes were attached pre-auricularly or over the temporal region over both hemispheres. Whether the optodes were placed preauricularly or temporally was not subject to a fixed standard but depended situationally on anatomical or other factors, as determined by the attending medical personnel. Unfortunately, the distance between the source-detector spacing was not documented during the data collection of the SIESTA trial [27]. Before applying the optodes, care was taken to ensure that no hair, foreign objects, or disruptive skin lesions (such as ulcers or sores) could interfere with the measurements. The attachments were then secured in place using adhesive tape. Continuous NIRS/BIS monitoring was defined as the implementation of the measurement electrodes/optodes immediately upon arrival in the angiography suite and before the intervention began, continuing until 5 min after the end of the procedure.

### Variables

Data on patient age, demographics, initial stroke severity (NIHSS), intracranial vessel with LVO, laterality of stroke, choice of sedation and degree of recanalization judged by the Thrombolysis in Cerebral Ischemia score (TICI) were extracted. Successful recanalization was defined as a TICI score of 2B or 3 [28, 29] Continuous NIRS and BIS monitoring was established, with recorded values documented every 5 min. The recorded NIRS values in this study are presented as tissue saturation index derived from oxy- and deoxyhemoglobin.

### Statistical analysis

We compared the initial NIRS and BIS values in a single measurement obtained upon arrival at the angiography suite with the values 5 min after successful or unsuccessful recanalization using a Wilcoxon signed rank test. We also initiated a subgroup analysis of a limited patient cohort with occlusions of the ICA, MCA in the M1 segment, MCA in the M2 segment, and a good reperfusion outcome, as judged by the TICI score (Thrombolysis in cerebral ischemia-Score).

Furthermore, we compared the NIRS and BIS values of the affected hemisphere between the first and second measurements and the difference in simultaneously recorded NIRS values between the two hemispheres before and after successful recanalization (dNIRS = NIRS unaffected hemisphere—NIRS affected hemisphere).

A further subgroup analysis examined whether the feasibility of using NIRS and BIS values for detecting recanalization would increase in cases with less-severely demarcated infarcts (ASPECTS score > 8) and occlusions (ICA, M1, M2) prior to EVT. Data from 19 patients with complete NIRS data and 18 patients with complete BIS data were evaluated under these criteria.

For the analysis of NIRS, dNIRS, and BIS in cases of unsuccessful recanalization (TICI < 2B), data from 10 patients with complete NIRS and dNIRS values and 8 patients with complete BIS values were available.

Primarily, this study did not differentiate between the chosen mode of sedation and analyzed patients treated with PS and GA together. To investigate the possibility that significant BIS values might be masked by the administration of anesthetics during general anesthesia, a subgroup analysis was conducted exclusively on patients who underwent mechanical recanalization under procedural sedation.

The statistical analysis of the NIRS and BIS values collected in the SIESTA trial [27] for mechanical recanalization in acute ischemic strokes of the anterior circulation was conducted using Excel (Microsoft Corporation. Microsoft Excel [Internet]. 2021. Available from: https://office.microsoft.com/excel) and GraphPad Prism (GraphPad Prism version 10.00 for Windows, GraphPad Software, San Diego, CA, USA, www.graphpad.com). Given the explorative nature of this post-hoc analysis, statistical test cannot be used to draw confirmatory conclusions. The reported p-values are to be understood as expository statistics. P-values were produces by Wilcoxon signed rank tests, the threshold for significance was set to 0.05. No adjustment for multiplicity was performed. The data were presented in boxplots and descriptive statistics in the form of median and interquartile range (IQR) were provided.

## Results

In the SIESTA trial [27], non-invasive neuromonitoring using NIRS and BIS was established in 150 patients with large vessel occlusions of the anterior circulation. Of the 150 patients in the SIESTA trial, 76 patients were included in the analysis. In comparison of included versus excluded patients, it was observed that excluded patients were initially more severely affected, exhibited a higher degree of demarcation, and tended to have more proximal vessel occlusions, although the rates of successful recanalization were similar (see Tables 1 and 2).

**Table 1:** Patient characteristics

	NIRS-monitoring	BIS-monitoring
N	76	58
Median age (IQR) – years	75.5 (64–84)	77 (66–84)
Sex – no. of women (%)	33 (43)	27 (47)
Hemisphere – no. of left (%)	40 (53)	30 (52)
Vessel occlusion – no (%)		
ICA + M1	25 (33)	20 (35)
M1	43 (57)	32 (55)
M2	8 (11)	6 (10)
Baseline NIHSS – median (IQR)	16 (14–20)	15 (14–19)
< 17	41 (54)	34 (59)
≥ 17	35 (46)	24 (41)
Baseline ASPECTS score		
< 8	21 (28)	13 (22)
≥ 8	55 (72)	45 (78)
Sedation – no. of PS (%)	34 (45)	25 (43)
Reperfusion result (TICI)– no. (%)		
1	6 (8)	5 (9)
2a	4 (5)	3 (5)
2b	39 (51)	24 (41)
3	27 (36)	25 (43)

Data are presented as counts in absolute numbers and percentage, if not stated otherwise. As a result of rounding, percentages may not exactly sum up to 100%

NIRS, near-infrared spectroscopy; BIS, Bispectral Index; IQR, interquartile range; ICA, internal carotid artery; M1, main branch of medial cerebral artery; M2, distal branch of medial cerebral artery; NIHSS, National Institutes of Health Stroke Scale; ASPECTS score. Alberta Stroke Program Early CT [computed tomographic] Score; PS, Procedural sedation; TICI, thrombolysis in cerebral infarction

**Table 2:** Comparison included and excluded patients

	Included	Excluded
N	76	74
Median age (IQR) – years	76.5 (66–84)	75 (61–81)
Sex – no. of women (%)	33 (43)	26 (35)
Hemisphere – no. of left (%)	41 (54)	46 (62)
Vessel occlusion – no (%)		
ICA + M1	28 (37)	33 (45)
M1	40 (53)	34 (46)
M2	8 (11)	7 (9)
Baseline NIHSS – median (IQR)	16 (14–20)	17 (15–20)
< 17	41 (54)	31 (42)
≥ 17	35 (46)	43 (58)
Baseline ASPECTS score		
< 8	21 (28)	34 (46)
≥ 8	55 (72)	40 (54)
Sedation – no. of PS (%)	34 (45)	32 (43)
Reperfusion result (TICI)– no. (%)		
1	6 (8)	5 (7)
2a	4 (5)	8 (11)
2b	39 (51)	28 (38)
3	27 (36)	33 (45)

Data are presented as counts in absolute numbers and percentage, if not stated otherwise. As a result of rounding, percentages may not exactly sum up to 100%

NIRS, near-infrared spectroscopy; BIS, Bispectral Index; IQR, interquartile range; ICA, internal carotid artery; M1, main branch of medial cerebral artery; M2, distal branch of medial cerebral artery; NIHSS, National Institutes of Health Stroke Scale; ASPECTS score. Alberta Stroke Program Early CT [computed tomographic] Score; PS, Procedural sedation; TICI, thrombolysis in cerebral infarction


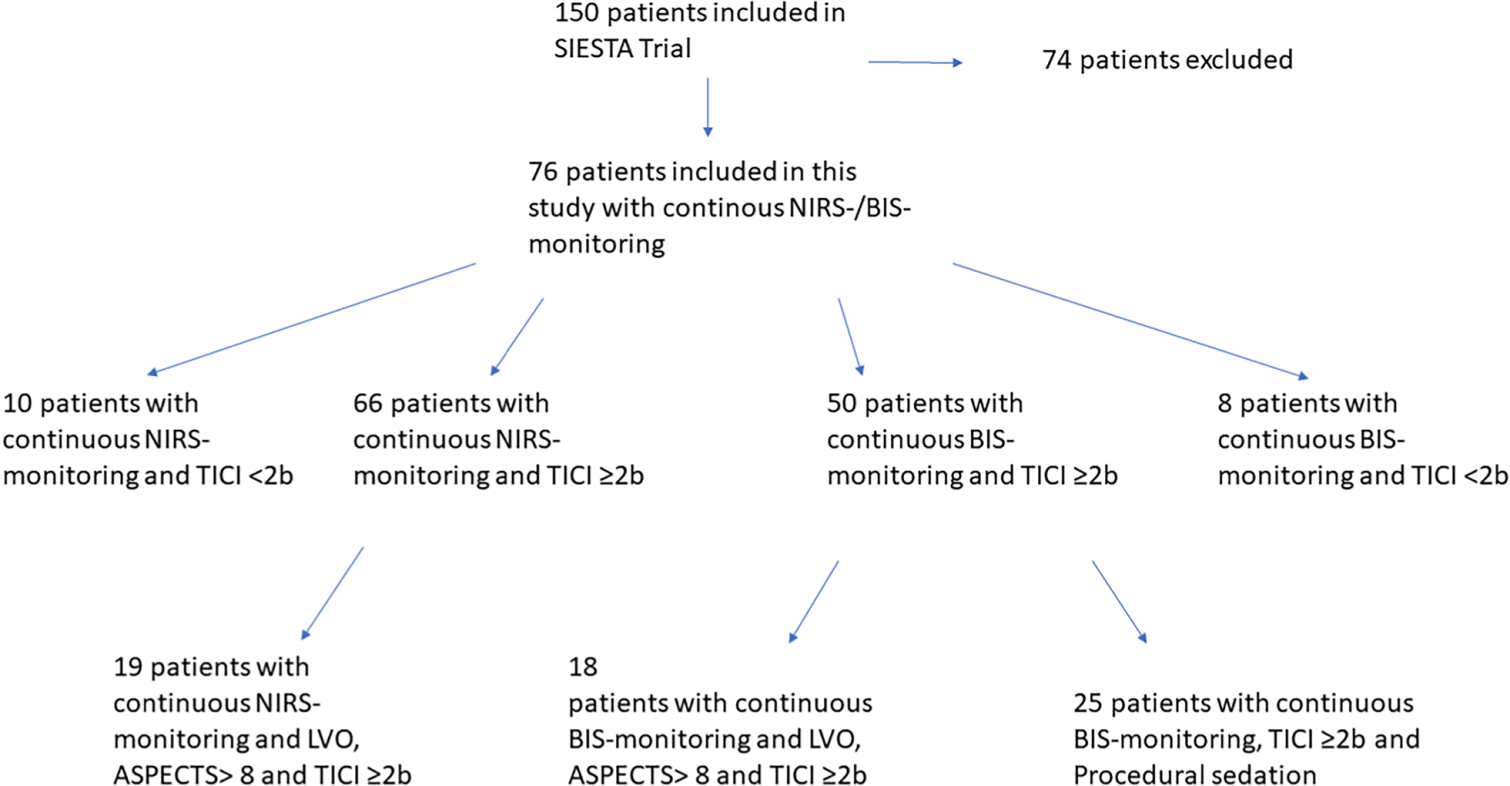


**Fig. 1:**
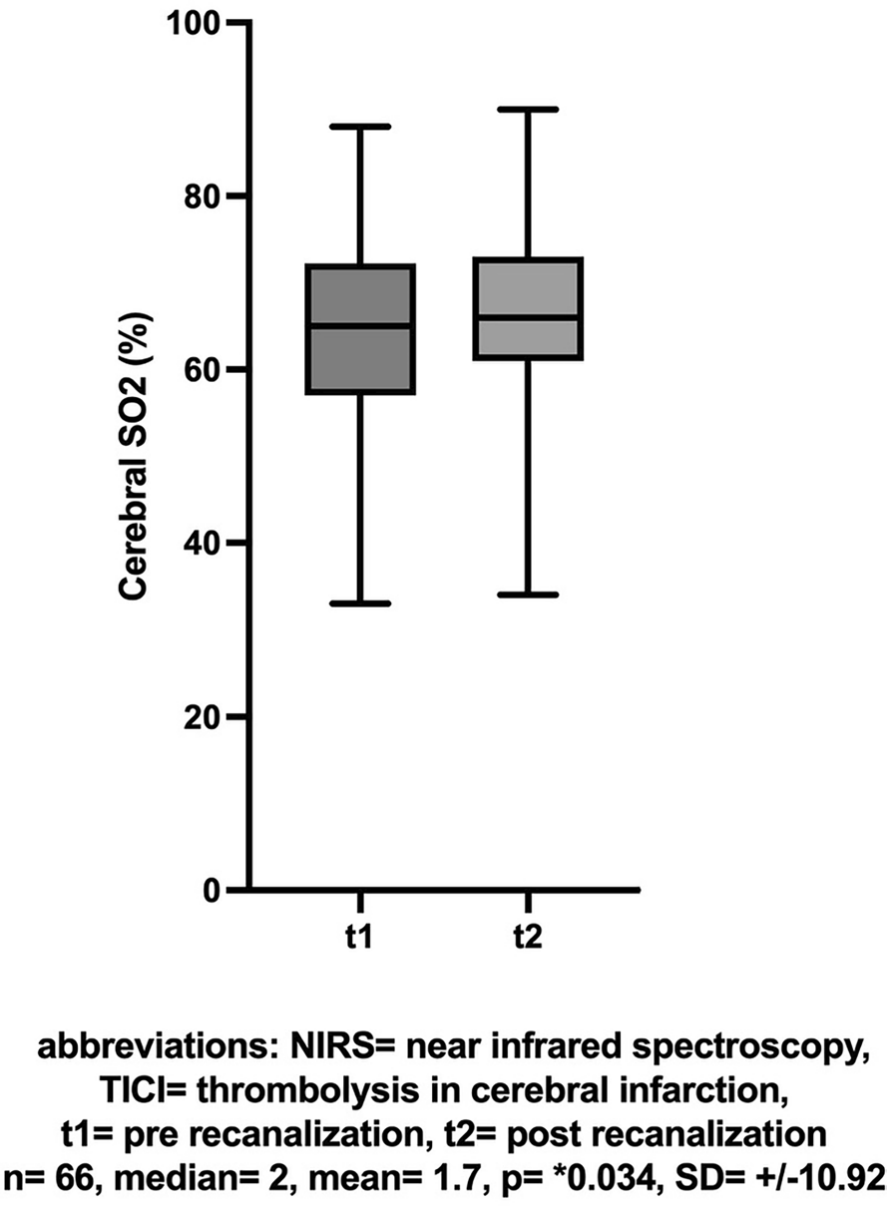
NIRS: Cerebral SO2 (%) before and after successful recanalization (TICI > 2b)

**Fig. 2:**
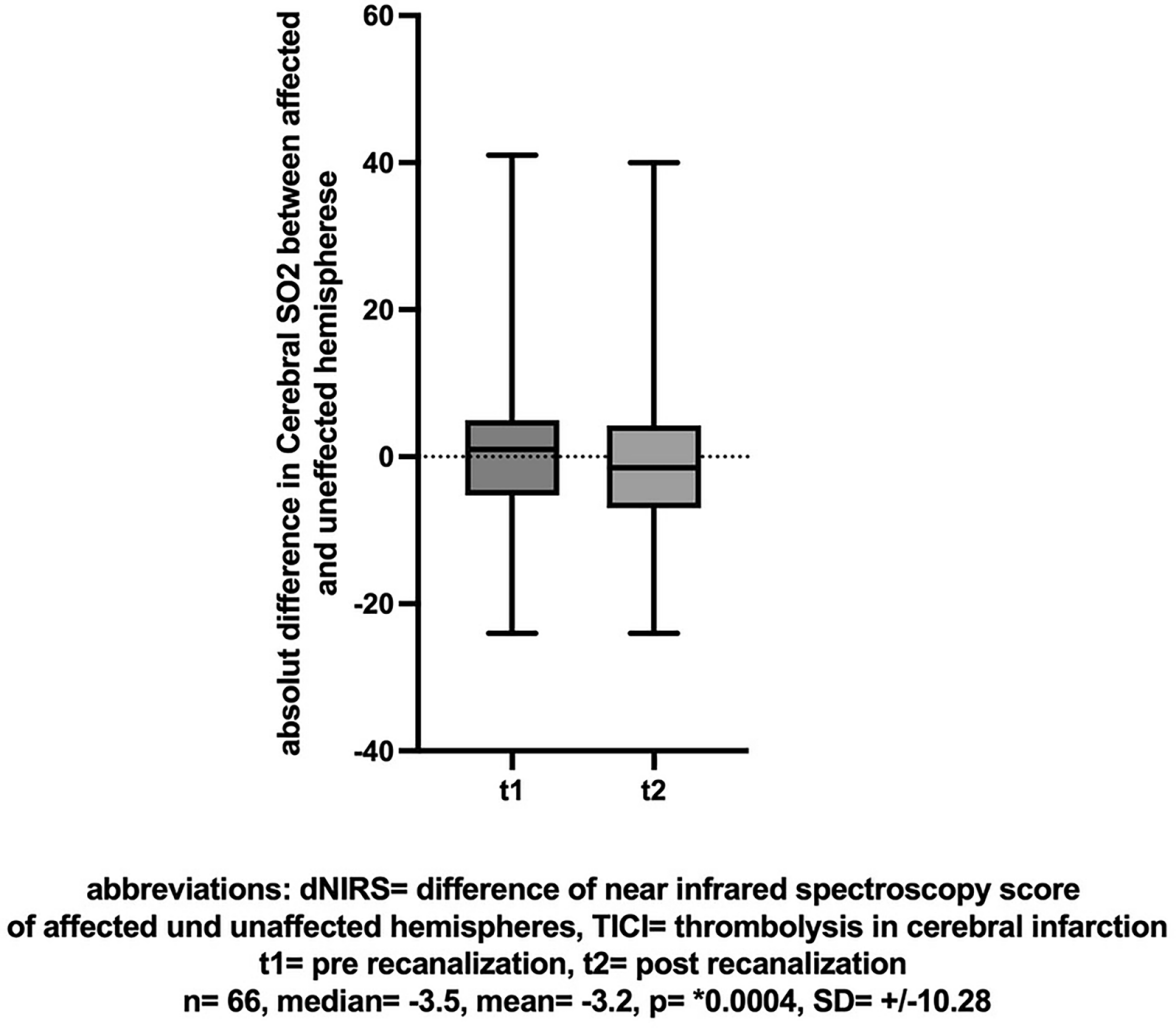
dNIRS-Score of affected und unaffected hemispheres before and after successful recanalization (TICI > 2b)

66 out of 76 patients with continuous NIRS monitoring achieved successful mechanical recanalization. In these patients there was an increase in the NIRS value (ergo regional oxygen saturation rSO_2_) of the affected hemisphere between baseline and post-recanalization (mean [± SD] change 1.7 [± 10.92], Cohen’s dz |*d*|= 0.156, *p = 0.034, insert Fig. 1 about here). The difference in NIRS values between the hemispheres also decreased between the first and second measurements (n = 66, mean [± SD] change  − 3.2 [± 10.28], Cohen’s dz |*d*|=  − 0.311, *p = 0.0004, insert Fig. 2 about here). The effect-size of both measurements showed only a small effect.

**Fig. 3:**
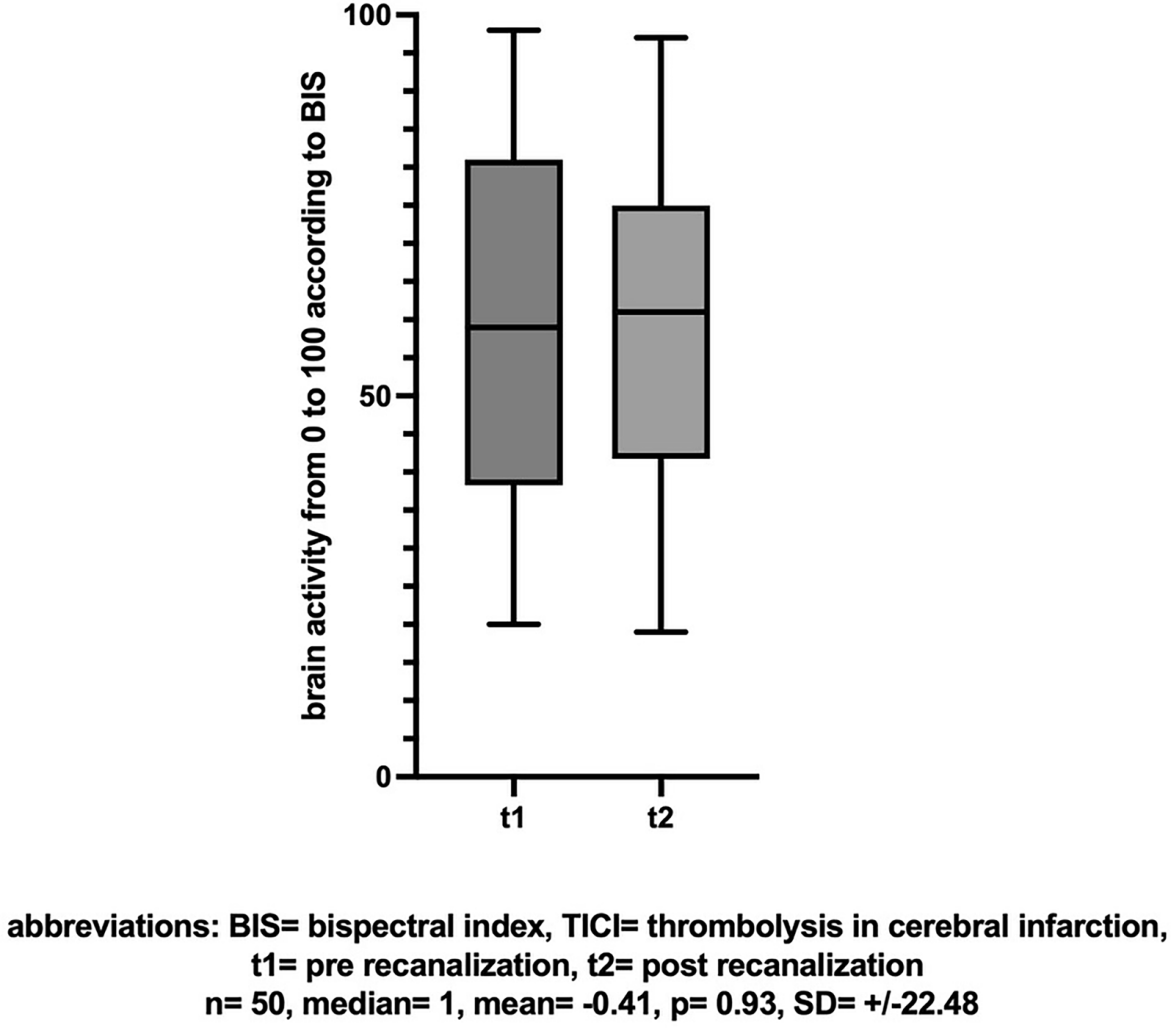
BIS-Score before and after successful recanalization (TICI > 2b)

50 out of 58 patients with continuous BIS monitoring achieved successful mechanical recanalization. But in contrast to the NIRS monitoring, the BIS monitoring did not show a difference between baseline and post-recanalization (n = 50, mean [± SD] change 1 [± 22.48], Cohen’s dz |*d*|= 0.044, *p* = 0.93, insert Fig. 3 about here).

**Fig. 4:**
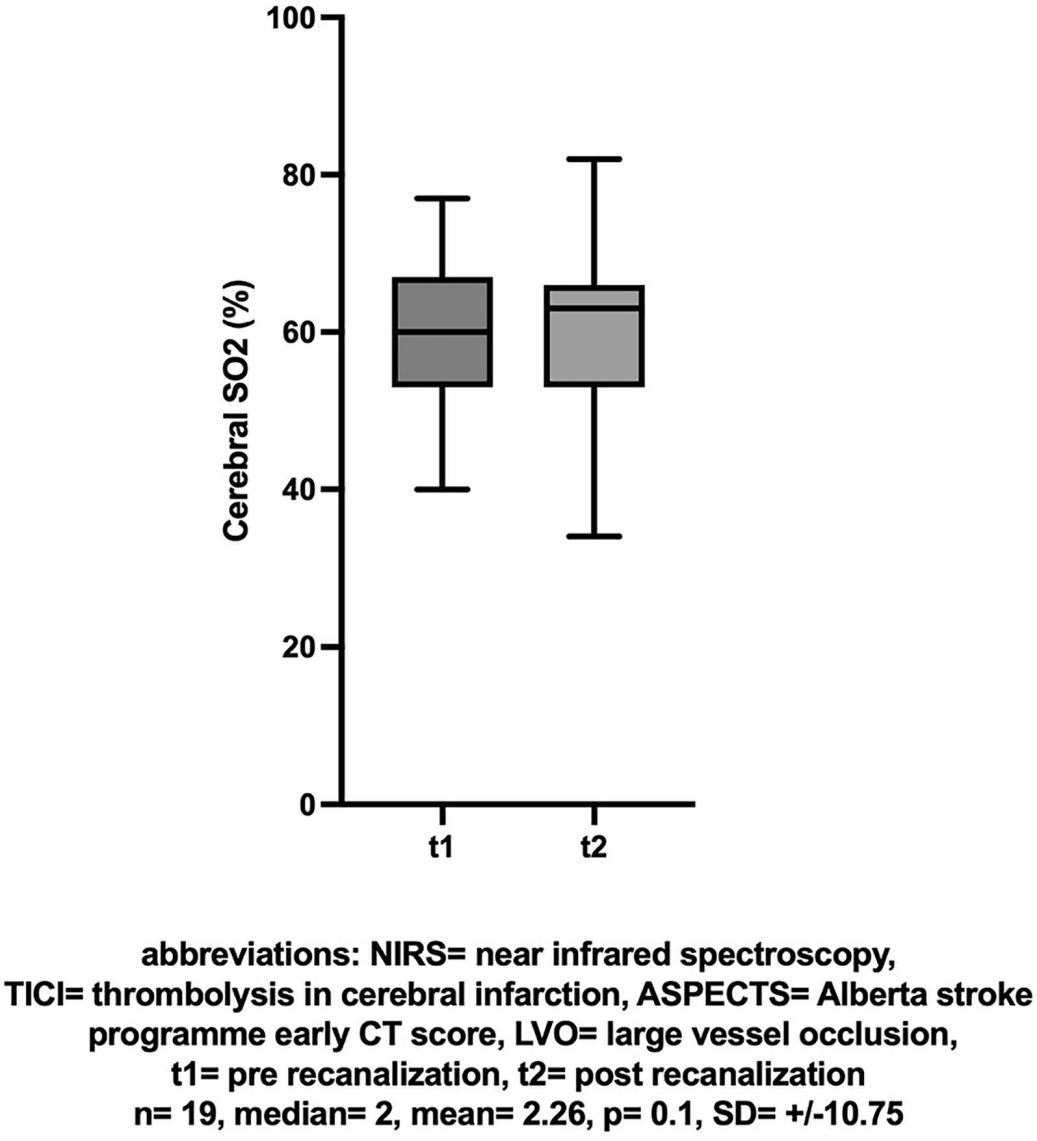
NIRS: Cerebral SO2 (%) before and after successful recanalization (TICI > 2b) of a large vessel occlusion (LVO) with little ischemic changes (ASPECTS > 8)

**Fig. 5:**
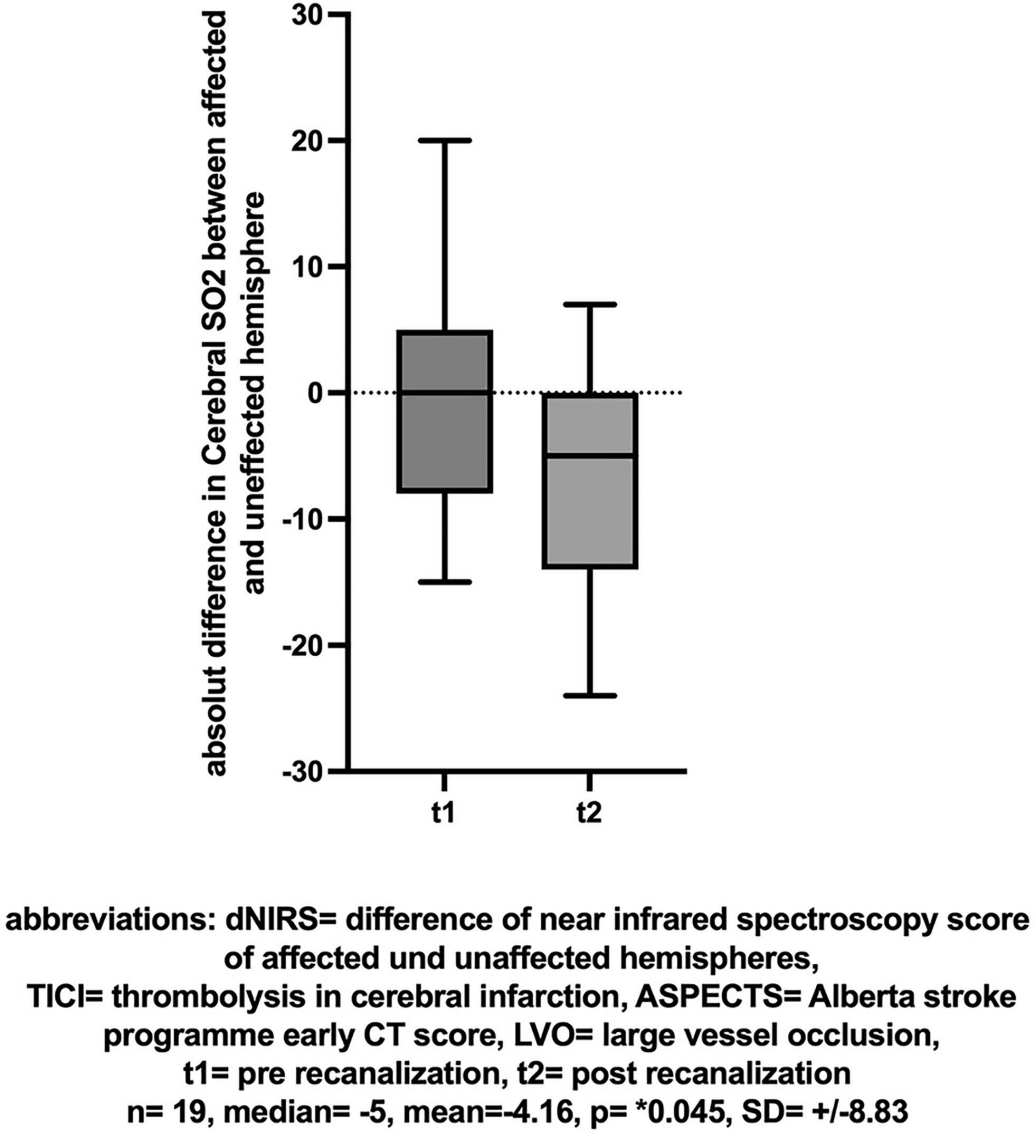
dNIRS-Score of affected and unaffected hemispheres before and after successful recanalization (TICI > 2b) of a large vessel occlusion (LVO) with little ischemic changes (ASPECTS > 8)

**Fig. 6:**
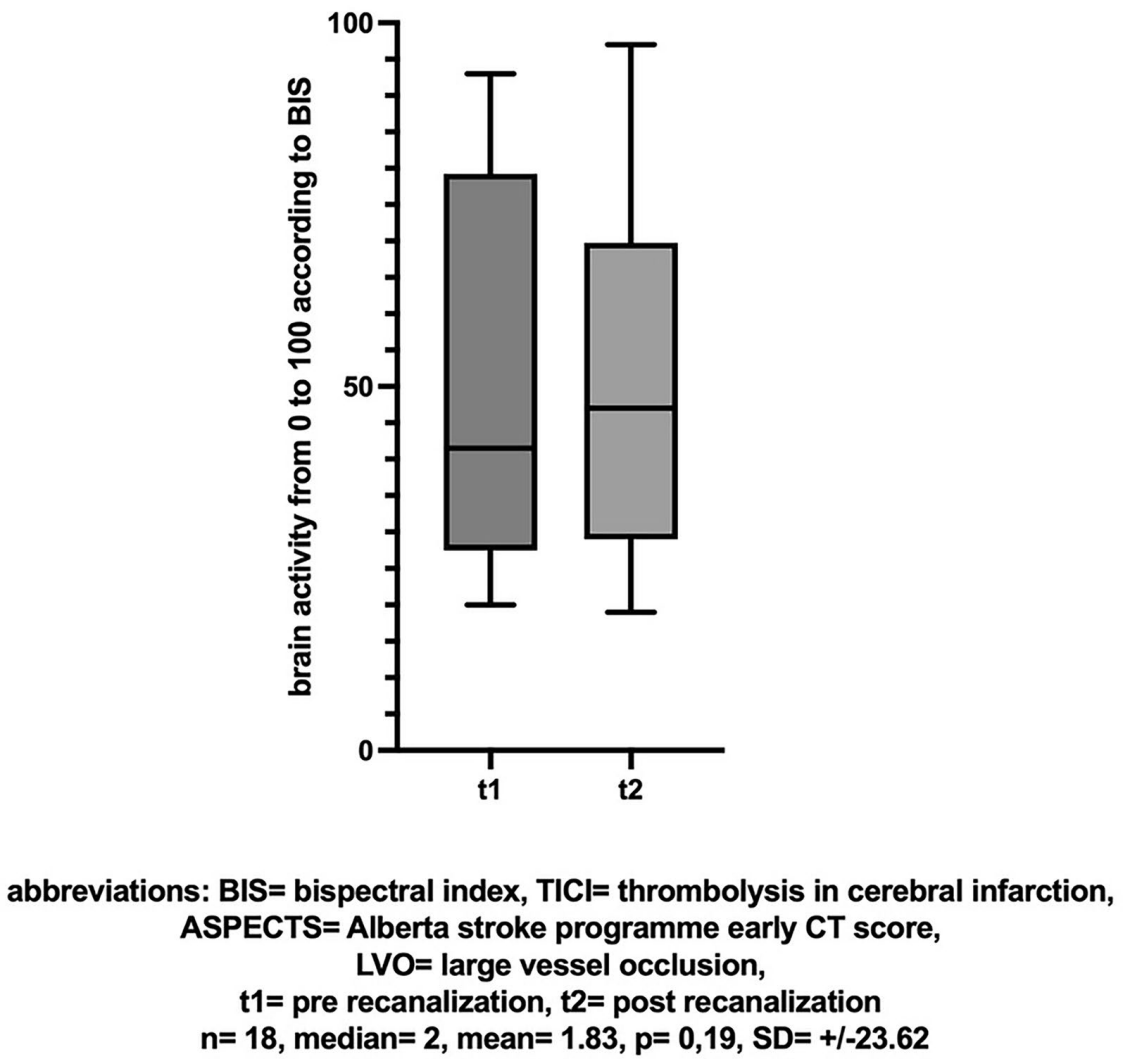
BIS-Score before and after successful recanalization (TICI > 2b) of a large vessel occlusion (LVO) with little ischemic changes (ASPECTS > 8)

In the subgroup of patients with occlusion at the ICA or M1 segment, an initial ASPECTS score > 8, and successful recanalization (TICI > 2b), a similar change in NIRS values was observed between the first and second measurements but it was not significant (n = 19, mean [± SD] change 2.26 [± 10.75], Cohen’s dz |*d*|= 0.21, *p* = 0.1, insert Fig. 4 about here). However, a comparison of dNIRS values showed a more pronounced difference (n = 19, mean [± SD] change  − 4.16 [± 8.83], Cohen’s dz |*d*|=  − 0.471, *p = 0.045, insert Fig. 5 about here). The effect-size showed a moderate effect. A comparison of BIS values in this subgroup did not yield differences (n = 18, mean [± SD] change, 1.83 [± 23.62], Cohen’s dz |*d*|= 0.077, *p* = 0.19, insert Fig. 6 about here).

**Fig. 7:**
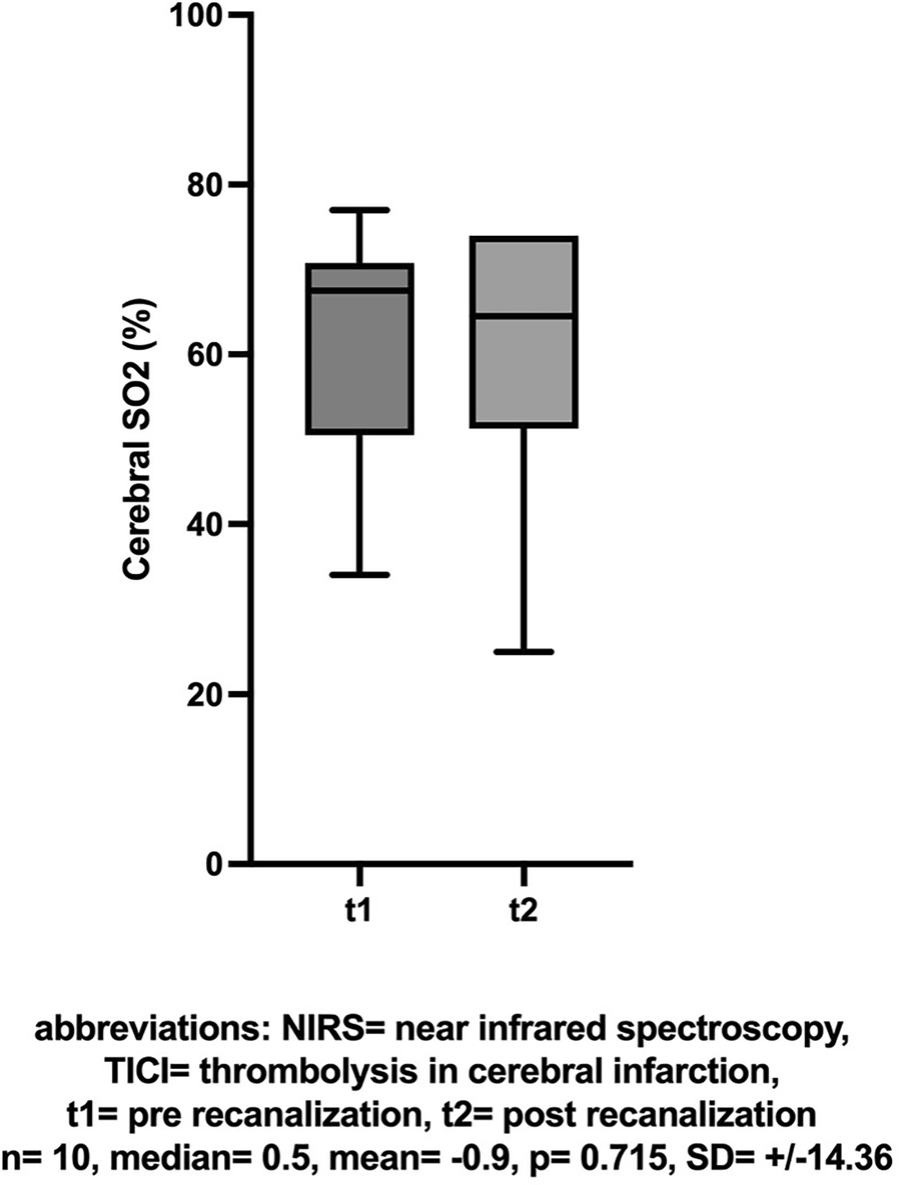
NIRS: Cerebral SO2 (%) before and after unsuccessful recanalization (TICI < 2b)

**Fig. 8:**
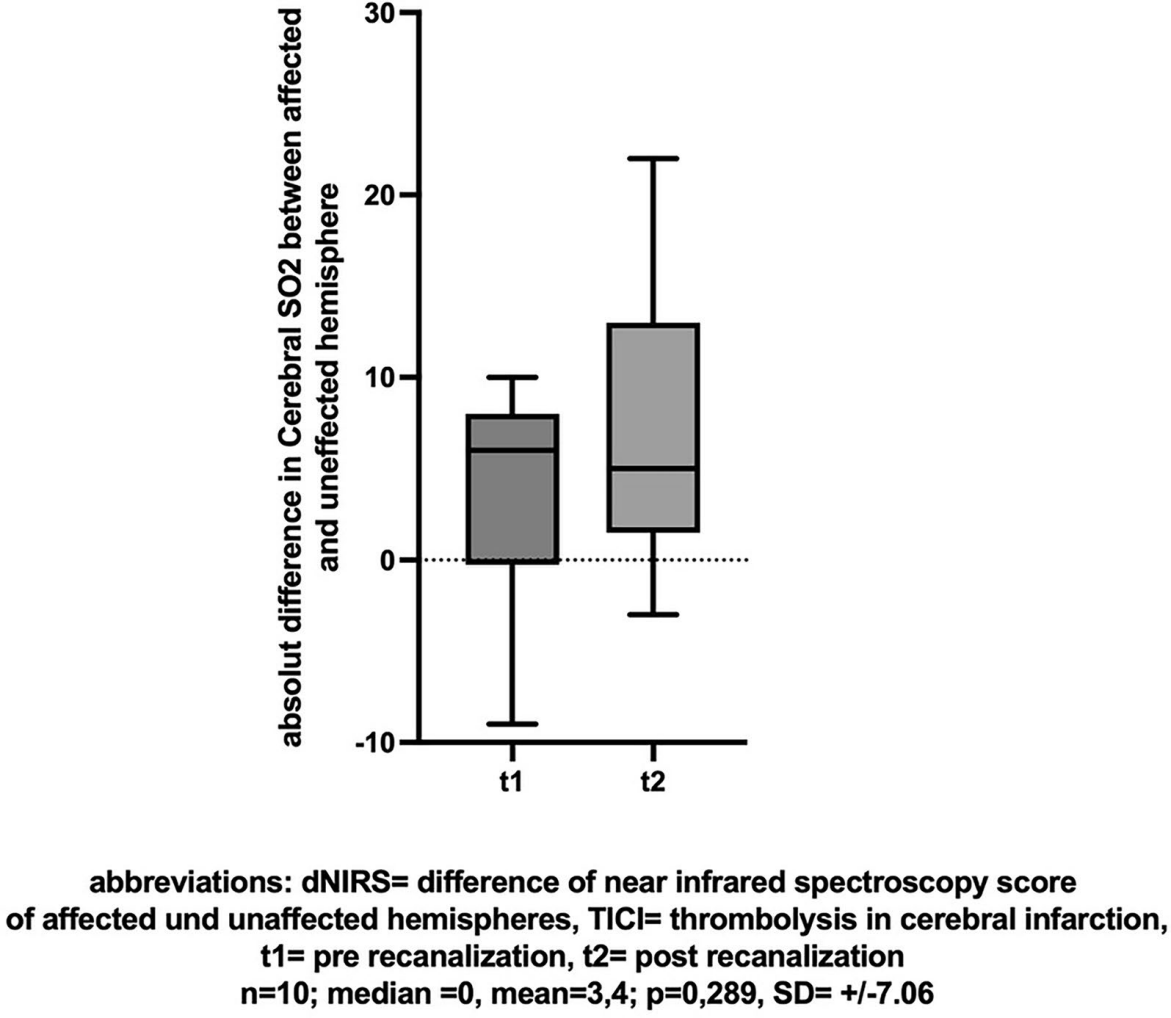
dNIRS-Score of affected and unaffected hemispheres before and after unsuccessful recanalization (TICI < 2b)

**Fig. 9:**
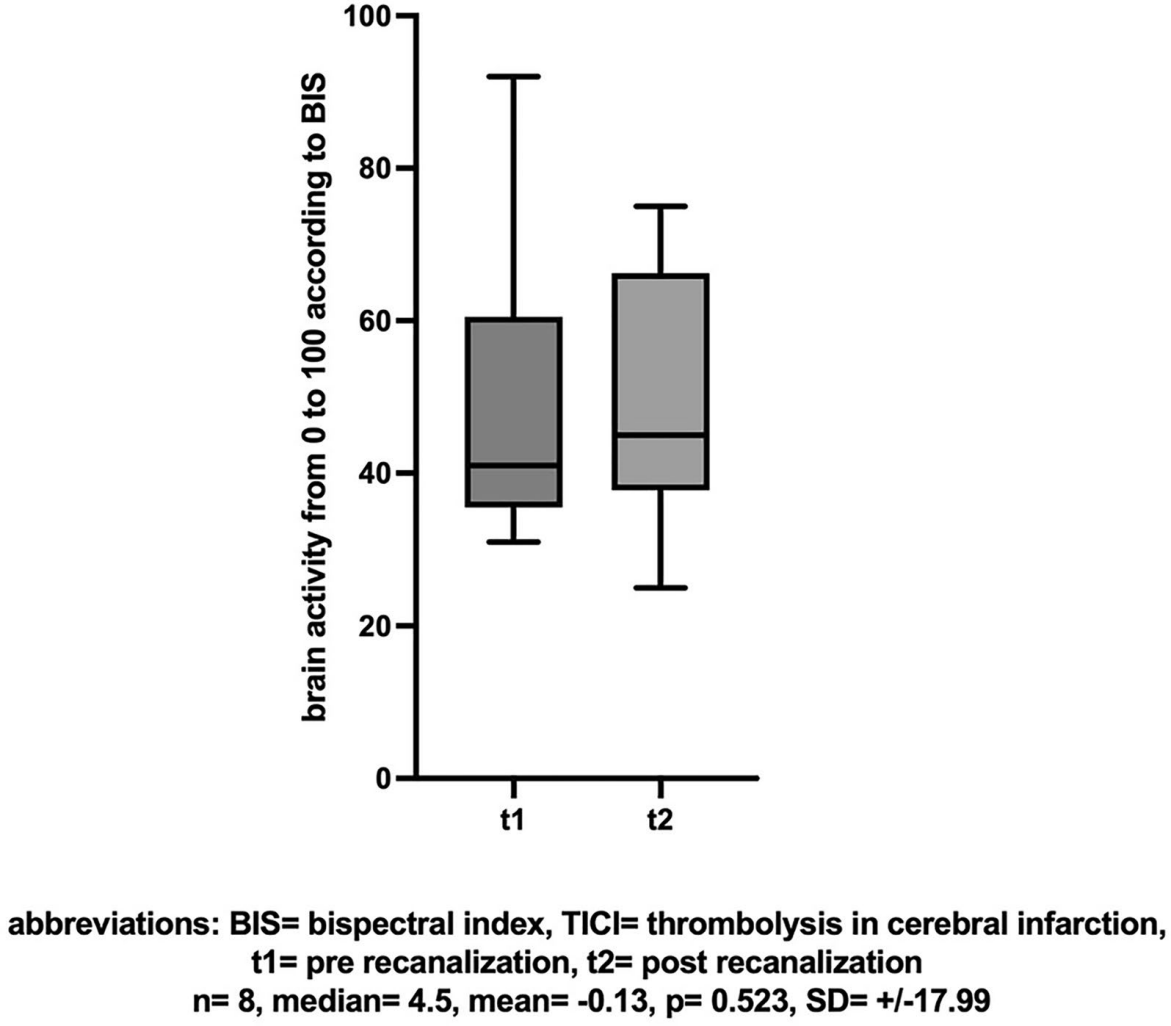
BIS-Score before and after unsuccessful recanalization (TICI < 2b)

In patients with unsuccessful recanalization (TICI < 2b), there was no difference in the comparison of NIRS values (n = 10, mean [± SD] change − 0.9 [± 14.36], Cohen’s dz |*d*|= − 0.063, *p* = 0.715, insert Fig. 7 about here), dNIRS values (n = 10, mean [± SD] change 3.4 [± 7.06], Cohen’s dz |*d*|= 0.482, *p* = 0.289, insert Fig. 8 about here), or BIS values (n = 8, mean [± SD] change − 0.13 [± 17.99], Cohen’s dz |*d*|= − 0.007, *p* = 0.523, insert Fig. 9 about here) pre- compared to post-recanalization.

**Fig. 10:**
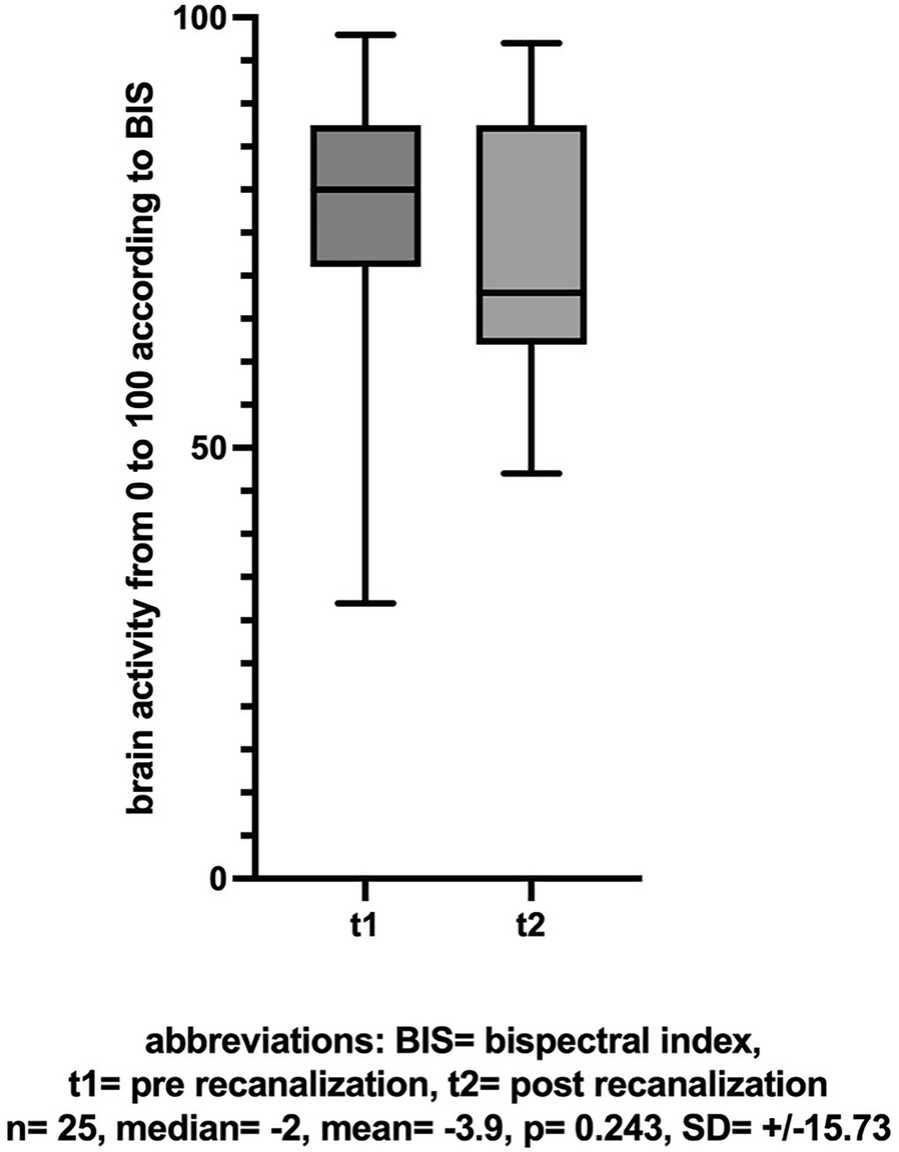
BIS-Score before and after recanalization performed in conscious sedation

A further analysis was conducted in the subgroup of patients who underwent EVT in conscious sedation, with successful recanalization (TICI > 2b), occlusion of the ICA, M1 and M2 segments, and without further specification of the ASPECTS score. No difference in BIS values between the first and second measurements was found in this analysis either (n = 25, mean [± SD] change  − 3.9 [± 15.73], Cohen’s dz |*d*|=  − 0.248, *p* = 0.243, insert Fig. 10 about here).

## Discussion

This study investigated the feasibility und utility of NIRS and BIS monitoring during EVT for AIS. Briefly, it demonstrates association of changes in NIRS values with successful reperfusion. Significant results were obtained from comparing the NIRS values of the affected hemisphere before and after recanalization, as well as from the difference in NIRS values between both hemispheres pre- and post-recanalization. It should be noted that the effect size in the comparison between NIRS and dNIRS data remained small; however, in the subgroup analysis restricted to LVO, ASPECTS > 8, and successful recanalization, the effect size was somewhat more pronounced, reaching a moderate level. In contrast, BIS monitoring did not yield any indicative changes.

Pathophysiologically, these non-invasive neuromonitoring methods measure different parameters.

NIRS utilizes the ability of near-infrared light to penetrate the skull in order to measure regional oxygen saturation (rSO_2_) using spectroscopic methods. However, the resulting signal consists of 70% venous signal and only 30% arterial signal [30]. Furthermore, signal interference from extracranial vessels can also influence the signal due to signal overlap [30]. This suggests that it is difficult to interpret absolute values of regional oxygen saturation; instead, relative changes in the measured regional oxygen saturation should be used as markers for changes in regional blood flow [31]. NIRS monitoring is applied, for example, during surgeries involving the internal carotid artery [19]. Our study demonstrates feasibility in using NIRS for intra-procedural neuromonitoring and a small improvement in NIRS-based rSO_2_ values as well as a decline in inter-hemispheric differences in rSO_2_, pre- and post-EVT, may indicate successful reperfusion.

BIS monitoring uses EEG signals to determine a dimensionless classification of the level of consciousness, with the algorithm underlying this classification being a proprietary secret. BIS monitoring is not currently used in the context of EVT; however, a study demonstrated a correlation between BIS values and functional outcomes in acute cerebral infarctions [32]. While BIS has not been described in this setting so far, previous studies on NIRS monitoring during EVT have yielded mixed results.

While many studies primarily utilize NIRS during the subacute phase of ischemic stroke [33, 34], few have investigated its application in the context of EVT [26, 35–37]. In a study involving three patients [26], significant results were observed; however, the included patients were clinically severely affected. This suggests that larger ischemic areas increase the likelihood that the established monitoring method covers the affected region. Studies with larger patient cohorts and inclusion of more distal occlusions [35–37] failed to replicate these positive findings, even though a more in-depth analysis in one study [35] revealed that lower rSO_2_ |diff values of the interhemispheric oxygen saturation difference at the end of the intervention and reduced variability in regional oxygen saturation (ASVrSO_2_) following the intervention were associated with increased mortality and poorer functional outcomes.

One potential explanation for the differing results has been attributed to variations in optode placement. However, an approach involving broad coverage, including frontal and temporal regions, also failed to yield positive outcomes [36]. Beyond the previously mentioned inaccuracies of NIRS monitoring due to a high proportion of venous signal contribution and potential interference from extracranial vessels [35, 36], regional oxygen saturation could also be influenced by compensatory mechanisms associated with robust collateral circulation. Nonetheless, stratification analyses provided no evidence to support this, particularly as the clinical presentation of ischemia in these patients suggests inadequate compensation by collaterals [36].

The positive findings of this study thus contrast with similarly sized investigations. Despite statistical significance, the absolute differences observed in the current cohort were small and the effect size ranged from small to moderate. A potential factor warranting further investigation is the adhesive material used for the optodes, which may represent an additional source of interference [36].

No significant changes between the two points of measurement in successful recanalization, successful recanalization of al large vessel occlusion and only little ischemic changes and in unsuccessful recanalization were obtained for BIS monitoring.

One of the limitations of this study is that only 66 out of the original 150 patients received continuous neuromonitoring with NIRS or BIS. This was primarily due to sensor displacement during the performance of intraprocedural flat panel CTs following successful recanalization, resulting in the inability to obtain values 5 min post-recanalization in a timely manner. A study design that ensures consistent data collection at the designated time points could certainly include more patients with continuous monitoring. Attempts to reposition the sensors were often unsuccessful, as the adhesive material lost its adhesion strength after displacement. Patient agitation, commonly observed in stroke patients, along with consequent hyperactivity and resistance to foreign objects applied to the body, may also explain the low rate of continuous monitoring measurements.

While BIS did not yield any indicative associations, the changes in NIRS monitoring associated with reperfusion may indicate clinical utility. The application of NIRS as a non-invasive neuro-monitoring tool for detecting intracranial vessel occlusions and their recanalization may positively extend the (momentary) information gained from angiography. In addition to the angiographic confirmation of recanalization, NIRS and BIS could enable earlier detection, potentially facilitating interventions such as earlier blood pressure reduction or periprocedural BP adjustments. However, this study must also assume that the practical benefit of non-invasive neuromonitoring is limited, as post-interventional NIRS-scores could be obtained in less than 50% of patients. Future studies should consider incorporating strategies to increase this proportion during the planning phase. Future studies could explore whether NIRS and BIS increases correlate with clinical outcomes or detect intraprocedural complications like hemorrhages or re-occlusions. Non-invasive neuromonitoring during mechanical recanalization offers user-friendly, portable, and continuous bedside monitoring, independent of clinical assessments. It is particularly useful during general anesthesia or when sedation impairs evaluation. Continuous monitoring may also track clinical deterioration from edema or hemispheric swelling more effectively than intermittent CT scans.

### Conclusion and future perspectives

Non-invasive neuromonitoring by NIRS could be a useful tool for continuous monitoring in acute ischemic stroke treatment, to ensure a continuous surveillance after successful recanalization. Focusing on the comparison of dNIRS values between the hemispheres appears most promising, as this yielded the highest effect size. Although the practical utility may be limited, as NIRS values could only be obtained in less than 50% of patients following successful thrombectomy in this study.

In this study, the NIRS method demonstrated changes associated with and hence indicative of recanalization. BIS monitoring, however, was not sensitive to changes during recanalization and could carry the risk of being masked by factors such as sedation. Hence, NIRS may hold the potential to detect vascular occlusions and their recanalization and should be further investigated in future studies, including the post-interventional phase.

## Data Availability

The datasets used and/or analysed during the current study are available from the corresponding author on reasonable request.
